# Optimized Thermal Treatment of Lithium‐Ion Battery Components as a Basis for Sustainable Pyrometallurgy

**DOI:** 10.1002/cssc.202501753

**Published:** 2025-10-14

**Authors:** Anna Pražanová, Jan Kočí, Jonáš Uřičář, Dominik Pilnaj, Daniel‐Ioan Stroe, Vaclav Knap

**Affiliations:** ^1^ Department of Electrotechnology Faculty of Electrical Engineering Czech Technical University in Prague Prague 16000 Czech Republic; ^2^ Department of Glass and Ceramics University of Chemistry and Technology Prague Technická 5 166 28 Prague Czech Republic; ^3^ Department of Polymers University of Chemistry and Technology Prague 16628 Prague Czech Republic; ^4^ Department of Energy Aalborg University Aalborg 9220 Denmark

**Keywords:** lithium‐ion battery, low‐temperature treatment, pyrometallurgy, recycling, surface changes, thermal decomposition

## Abstract

The escalating global demand for lithium‐ion batteries necessitates efficient and sustainable end‐of‐life management. Major recycling routes such as pyrometallurgy and hydrometallurgy offer promising paths for metal recovery, but their efficiency often depends on the pretreatment of spent batteries. However, optimizing low‐temperature pretreatment for complete organic removal while preserving active material integrity remains challenging. This study investigated thermal decomposition and surface changes of key battery components—lithium nickel manganese cobalt oxide (NMC622) cathode, graphite anode, and polymeric separator—from 100 to 800 °C, focusing on the 400–650 °C industrial interval. Material responses were characterized using thermo‐gravimetric analysis coupled with mass spectrometry, isothermal mass loss, and scanning electron microscopy with energy‐dispersive X‐ray spectroscopy. A 500 °C treatment was identified as optimal, enabling complete organic carbon removal within 1 h without compromising the NMC spinel structure or current collector degradation. This precise control reduces energy consumption and mitigates hazardous gas release, enhancing environmental sustainability and providing a practical, scalable, and cost‐effective strategy for improving battery recycling. These findings help to define the parameters for efficient electroactive material separation. This work advances the understanding of low‐temperature thermal pretreatment for battery recycling, supporting a circular economy for critical materials.

## Graphical abstract

This study investigated thermal decomposition and surface changes of lithium nickel manganese cobalt oxide (NMC) cathode, graphite anode, and polymeric separator from 100 to 800 °C, focusing on the 400–650 °C industrial range. A 500 °C treatment was optimal, removing all organic carbon within 1 h while preserving the NMC spinel structure and preventing current collector degradation.

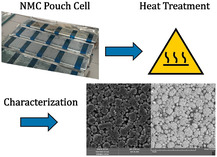

## 1 Introduction

The escalating global demand for lithium‐ion batteries (LIBs), especially in electric vehicles (EVs), necessitates efficient and sustainable end‐of‐life (EOL) management strategies.^[^ ^1^, ^2^ ^]^ In parallel, growing environmental concerns and the limited availability of critical raw materials such as lithium, cobalt, and nickel underscore the importance of developing circular economy solutions that support resource security and industrial resilience.^[^ ^3^, ^4^ ^]^

Regardless of the final metallurgical recovery route, the recycling of spent LIBs typically begins with a series of pretreatment steps, among which thermal treatment is critical for handling the organic components^[^ ^5^, ^6^, ^7^ ^]^ This step is foundational for both major recycling pathways. For hydrometallurgical processes, a controlled low‐temperature treatment (typically up to 800 °C) is employed to decompose the organic binders (e.g., polyvinylidene fluoride ‐ PVDF) and polymeric separators. This liberates the active materials from the current collectors, making them accessible for efficient downstream chemical leaching^[^ ^8^, ^9^, ^10^ ^]^ For pyrometallurgical processes, this low‐temperature stage serves a similar purpose of removing volatile and flammable organic materials to ensure safer and more stable operation of the subsequent high‐temperature smelting (1500–1800 °C) for metal recovery.^[^ ^11^, ^12^ ^]^ It should be noted, however, that some industrial pyrometallurgical operations perform direct smelting of battery packs without a separate low‐temperature pretreatment step.^[^ ^13^, ^14^ ^]^ Our work focuses on optimizing the separate, low‐temperature pretreatment, as this approach offers better control over organic degradation and preserves the integrity of active materials, enhancing the efficiency and sustainability of the overall recycling chain.

The first stage conditions the internal electrode–separator assembly and mitigates safety hazards from residual electrolytes and flammable materials.^[^ ^15^ ^]^ This step is often decoupled from metallurgical recovery to control organic degradation better, preserve active materials’ integrity, and facilitate delamination for downstream recovery. However, decomposition involves overlapping reactions with specific onset temperatures, requiring precise process control. Among various chemistries, lithium nickel manganese cobalt oxide (NMC) is widely studied due to its prevalence in high‐energy cells and complex thermochemical behavior.^[^ ^16^ ^]^ Industrially, external casings (polymer‐laminated aluminum (Al) or nickel) are removed before thermal treatment, so research focuses on the internal stack: cathode, anode, and separator.^[^ ^15^, ^17^ ^]^

NMC cathodes are generally thermally stable relative to organic compounds, but their behavior depends on delithiation and residual binders or salts.^[^ ^18^ ^]^ While the spinel structure may persist up to 800 °C,^[^ ^19^ ^]^ side reactions involving lithium salts (e.g., lithium hexafluorophosphate, LiPF_6_; lithium fluoride, LiF; lithium carbonate, Li_2_CO_3_) can lead to the release of carbon dioxide (CO_2_) or hydrogen fluoride (HF) and phosphorus‐containing gases.^[^ ^20^, ^21^ ^]^ Furthermore, the Al current collector, with a melting point of ≈660 °C, may melt and encapsulate active material if temperatures exceed this threshold,^[^ ^22^, ^23^ ^]^ compromising downstream separation and material quality.^[^ ^12^, ^24^, ^25^ ^]^ Therefore, precise temperature control is critical to remove organic compounds while preserving cathode integrity.^[^ ^26^ ^]^

Graphite anodes exhibit high thermal stability under inert atmospheres but oxidize in air above ≈500 °C, resulting in significant CO_2_ release and mass loss.^[^ ^27^ ^]^ Complete combustion typically occurs between 600 and 700 °C, depending on oxygen content in the atmosphere, heating rate, and surface area^[^ ^27^, ^28^, ^29^ ^]^ Residual surface compounds, such as lithium alkyl carbonates or Li_2_CO_3_, originating from solid electrolyte interphase layer degradation, may also affect the oxidation pathway and initiate earlier decomposition.^[^ ^30^ ^]^

Polymeric separators, typically made of polyethylene (PE) or polypropylene (PP), begin to degrade below 200 °C. This involves initial melting followed by decomposition into hydrocarbon species^[^ ^31^, ^32^, ^33^ ^]^ Polyvinylidene fluoride (PVDF), commonly used as a binder, decomposes between 350 and 500 °C, releasing HF, water, and low‐molecular‐weight fragments.^[^ ^34^ ^]^ Above 500 °C, remaining residues are mostly converted into stable inorganic phases; for example, alumina‐coated separators may yield aluminum oxide (Al_2_O_3_) after treatment.^[^ ^33^ ^]^

Given the complexity of multicomponent battery architectures, most studies on low‐temperature pyrometallurgical pretreatment have focused on achieving efficient organic removal while preserving valuable materials, as both are essential for scalable LIB recycling^[^ ^35^, ^36^, ^37^, ^38^ ^]^ Thermal exposure between 500 and 600 °C is widely regarded as a critical step for decomposing polymeric binders and evaporating electrolyte residues, thereby improving the efficiency of mechanical comminution and downstream metal recovery.^[^ ^37^ ^]^ However, temperatures above 600 °C can lead to the embrittlement or melting of Al foils, complicating their separation. In parallel, elevated temperatures also promote the carbothermic reduction of active materials, such as LiCoO_2_, which begins decomposing around 700 °C and affects both mass loss and lithium recovery yields. Although reviews of pretreatment strategies confirm that calcination between 150 and 650 °C efficiently removes conductive carbon and organic compounds, reducing binder adhesion and enhancing separability, they also emphasize challenges related to high energy demands and the release of hazardous gases, including toxic fluorinated species.^[^ ^35^, ^37^ ^]^ While several studies have explored high‐temperature pyrometallurgy, a comprehensive insight into low‐temperature pretreatment effects on cathode and separator integrity remains limited. Therefore, a detailed, component‐specific understanding of thermal degradation, particularly how controlled heating influences the structure, surface chemistry, and separability of individual active materials and polymeric separators, is crucial for the development of energy‐efficient, scalable recycling processes. Such processes must not only ensure effective organic removal but also minimize unwanted side reactions and preserve material functionality.^[^ ^38^, ^39^ ^]^

While several studies have explored high‐temperature pyrometallurgy, a comprehensive insight into low‐temperature pretreatment effects on cathode and separator integrity remains limited. Therefore, the primary aim of this work is to systematically investigate the thermal degradation and surface evolution of individual critical components—the NMC622 cathode, graphite anode, and polymeric separator—to identify an optimal pretreatment temperature. The novelty of this study lies in its integrated analytical approach. We combine dynamic analysis via thermogravimetric analysis coupled with mass spectrometry, static isothermal mass loss studies, and detailed surface characterization using scanning electron microscopy with energy‐dispersive X‐ray spectroscopy. This allows us to bridge the gap between simple mass loss, and a deeper understanding of how controlled heating influences the structural integrity, surface chemistry, and separability of materials. By defining a precise, energy‐efficient temperature (500 °C) that ensures complete organic removal while preserving the valuable inorganic components, this work provides practical, foundational data for designing and scaling up sustainable pretreatment processes applicable to any subsequent metallurgical recovery, with a particular focus on its role as a basis for sustainable pyrometallurgy.

## 2 Experimental Section

### 2.1 Material

The LIB pouch cells used in this study possess a nominal voltage of 3.65 V and a rated capacity of 78 Ah. These cells were previously characterized in our previous work by Pražanová et al.^[^ ^40^ ^]^ Each cell comprises 36 stacked layers comprising NMC622 cathode materials, where the numbers indicate the molar ratio of Ni:Mn:Co, a polymer separator, and graphite‐based anode layers. The electrolyte consists of LiPF_6_ in an organic solvent, and the entire assembly was encapsulated in a flexible polymer–aluminum laminated pouch.

**Figure 1:**
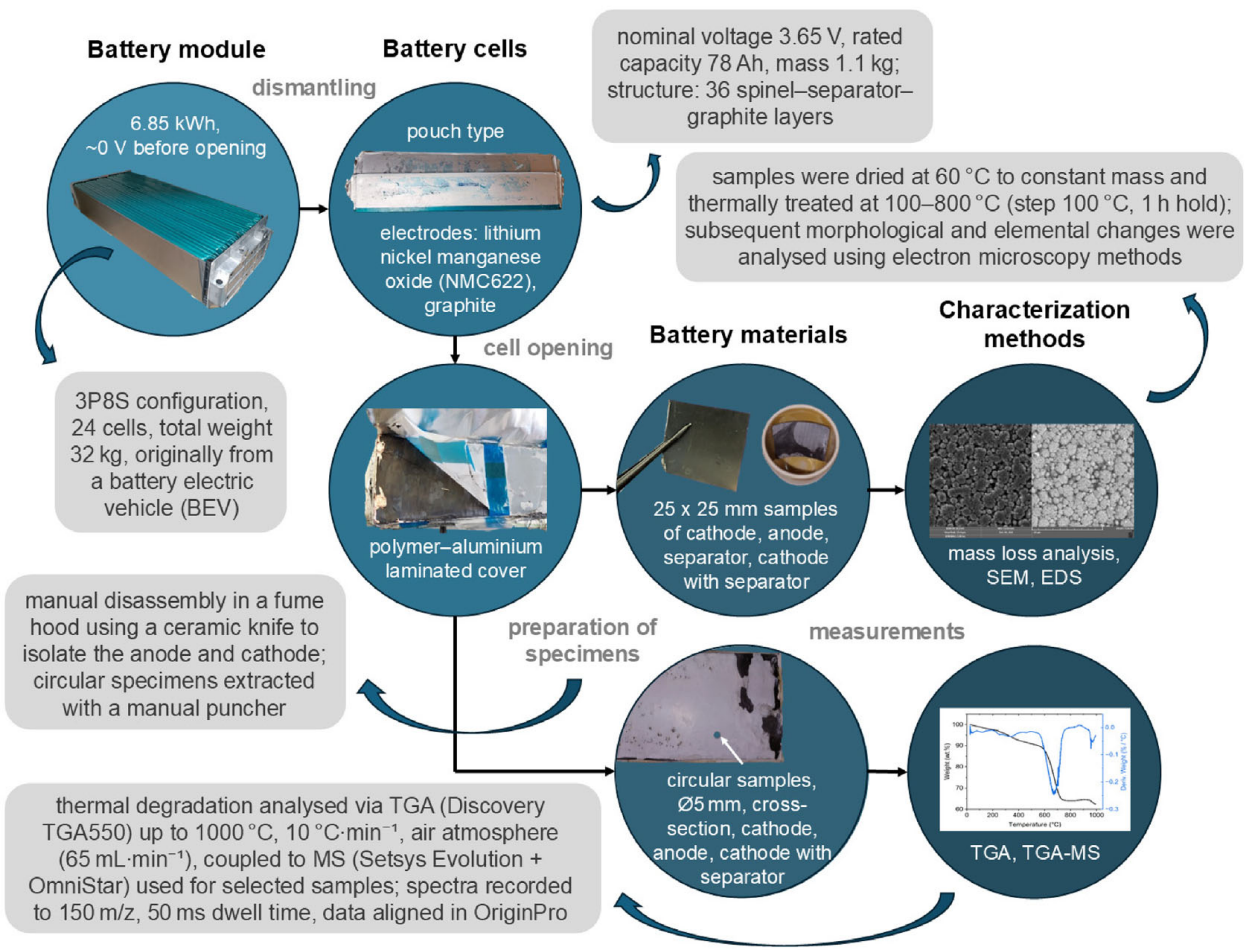
Overview of the experimental workflow: opening of the battery module, extraction and disassembly of pouch cells, preparation of specimens of battery materials, and subsequent morphological and elemental analyzes (SEM; energy‐dispersive X‐ray spectroscopy, EDS) followed by thermal analyzes (thermogravimetric analysis, TGA; thermogravimetric analysis coupled with mass spectrometry, TGA‐MS).

The module was sourced from an electric vehicle at its EOL, representing a realistic feedstock for recycling. It had a nominal voltage of 29.36 V and a total energy capacity of 6.85 kWh. While the precise State of Health (SOH) was not determined, it was assumed to be in the typical 70%–80% range for an EOL battery. Critically, for safety reasons, before disassembly, the module was deeply discharged to ≈0 V. This process subjects the cell components to harsh conditions known to induce further irreversible degradation, thus positioning our samples as a challenging but representative case for thermal treatment. It could be postulated that a process proven effective on this degraded material will be equally or more successful on less degraded batteries (higher SOH). To access the internal cells, the module's casing was carefully milled open, and the adhesive bonding was weakened by applying alcohol to facilitate their separation. Subsequently, the cells were manually extracted from the opened module. The open structure of the module, individual battery cells, their opening procedure, and specimen preparation and characterization are shown in **Figure** **1**.

### 2.2 Methods

#### 2.2.1 Specimen Preparation

A set of samples consisting of the active material coated on its native current collector was prepared for analysis. This configuration was intentionally maintained because a primary objective of the study is to optimize a thermal process that facilitates the separation (delamination) of the active material from the foil by decomposing the binder. Analyzing the isolated powder without the foil would therefore not align with the study's practical, recycling‐oriented goals. To ensure statistical validity and reproducibility, all measurements were conducted in five independent replicates. The battery cell was carefully disassembled using a ceramic knife, and individual components, specifically the cathode enclosed within the separator pouch and the anode, were isolated. All procedures were conducted inside a laboratory fume hood.

Following disassembly, as described in Figure 1, intact electrodes and the separator layers were predried at ambient temperature to evaporate the carbonates. The predried layers were subsequently sectioned into 25 × 25 mm square samples using a precision paper cutter. Afterwards, the sections were dried at 60 °C until no further decreases in mass were observed (approximately 1 week). The final samples were weighed in porcelain crucibles using an analytical balance.

The specimens were classified into three groups according to their functional role within the cell: cathode, anode, and separator. In addition, a fourth reference group was prepared, consisting of the cathode layer covered by a separator on both sides, to control potential interfacial effects. This configuration deviates from the standard architecture, in which separators on both sides typically enclose the cathode. Five independent replicates were measured for each sample category to ensure statistical validity.

For the thermal degradation analyzes, circular samples with a diameter of 5 mm were punched from an unopened battery cell using a manual puncher. These specimens comprised the entire cross‐section of the battery cell, including spinel, separator, and graphite layers, and are hereinafter referred to as “cross‐section samples.” In addition to the full cross‐section samples, a separate specimen of cathode, anode, and the cathode with a two‐sided separator was explicitly prepared for TGA‐MS measurements.

#### 2.2.2 Material Characterization Methods

##### 2.2.2.1 Thermogravimetric Analysis

Thermal degradation in dynamic mode was assessed using a thermogravimetric analyzer (Discovery TGA550 Auto Advanced, TA Instruments, USA). A cross‐section specimen, including all electrochemically active layers with a weight of ≈25 mg, was subjected to heating from room temperature (24 °C) to 1000 °C at a constant heating rate of 10 °C min^−1^ in an air atmosphere with a flow rate of 65 mL min^−1^.

##### 2.2.2.2 Thermogravimetric Analysis – Mass Spectrometry

The chemical composition of degradation products was investigated using coupled TGA‐MS. The following sample types were analyzed: cross‐section, anode, cathode, and cathode with one‐sided separator. Measurements were performed on a TG‐DTA Setsys Evolution system (Setaram, France) coupled to an OmniStar quadrupole mass spectrometer (Pfeiffer Vacuum, Germany).

Before thermal treatment, all samples were stabilized at 30 °C for 30 min. Subsequently, the samples were heated in an air atmosphere at a constant heating rate of 10 °C min^−1^. After the heating and thermogravimetric data acquisition were completed, the samples were allowed to cool under ambient conditions, while mass spectrometric data acquisition continued for an additional 50 min. Mass spectra were recorded up to 150 m z^−1^ (mass‐to‐charge ratio) with a resolution of 1 m z^−1^; each spectrum was acquired over 8 s, corresponding to a dwell time of 53 milliseconds per m/z unit. The data obtained from TGA and MS were synchronously aligned, processed, annotated, and visualized using OriginPro software. Representative ions corresponding to specific compounds or chemical groups were selected based on the NIST17 library of electron ionization spectra.

##### 2.2.2.3 Static Thermal Mass Loss Analysis

Samples were placed in a CLASIC laboratory furnace with a front‐opening design for static thermogravimetric measurements. Heating was performed at 5 °C min^−1^ to target temperatures of 100, 200, 300, 400, 500, 600, 700, and 800 °C, each followed by a 1h isothermal hold, then cooled to room temperature by natural convection overnight. The relatively high heating rate and short exposure time were chosen to reduce the required time to effectively obtain the studied materials, which corresponds to the study's objectives and the economic demands of industrial use. This approach is relevant for pyrometallurgical pretreatment in battery recycling, where energy efficiency and time optimization are critical. Temperatures were selected to identify the minimum effective recycling threshold under economic constraints, focusing in detail on the 400 to 650 °C range, with measurements at 400, 450, 500, 550, 600, and 650 °C.

After cooling to ambient temperature under natural conditions, gravimetric measurements were performed using a calibrated KERN analytical balance to evaluate mass loss resulting from thermal exposure. Each sample type was measured in five independent replicates, and the mass was recorded to four decimal places to ensure high precision. This methodology enabled a comprehensive assessment of thermally induced mass changes, enhancing understanding of material decomposition behavior across various temperature conditions.

##### 2.2.2.4 Scanning Electron Microscopy

Following thermal exposure, the samples were subjected to structural and compositional analyzes to assess changes induced by heat treatment. SEM was employed to examine modifications in surface morphology using a TESCAN VEGA 3 LMU scanning electron microscope. Imaging was conducted at an accelerating voltage of 20 kV, a working distance of 15 mm, and a beam intensity of 15 nA, utilizing both secondary and backscattered electrons at a magnification of 2000 ×.

To complement the morphological investigation, EDS was performed to determine alterations in elemental composition. Measurements were conducted using an OXFORD Instruments INCA 350 EDS analyzer, enabling precise chemical microanalysis of the observed samples. Data were processed using AZtec software (version 4.4), with composition measurements taken over an area of 0.005 mm^2^, yielding an accuracy of ± 0.1 % for each measured point. Micro‐X‐ray elemental mapping was also carried out to visualize the spatial distribution of elements across the sample surface.

## 3 Results and Discussion

### 3.1 Dynamic Thermal Degradation Analysis

**Figure 2:**
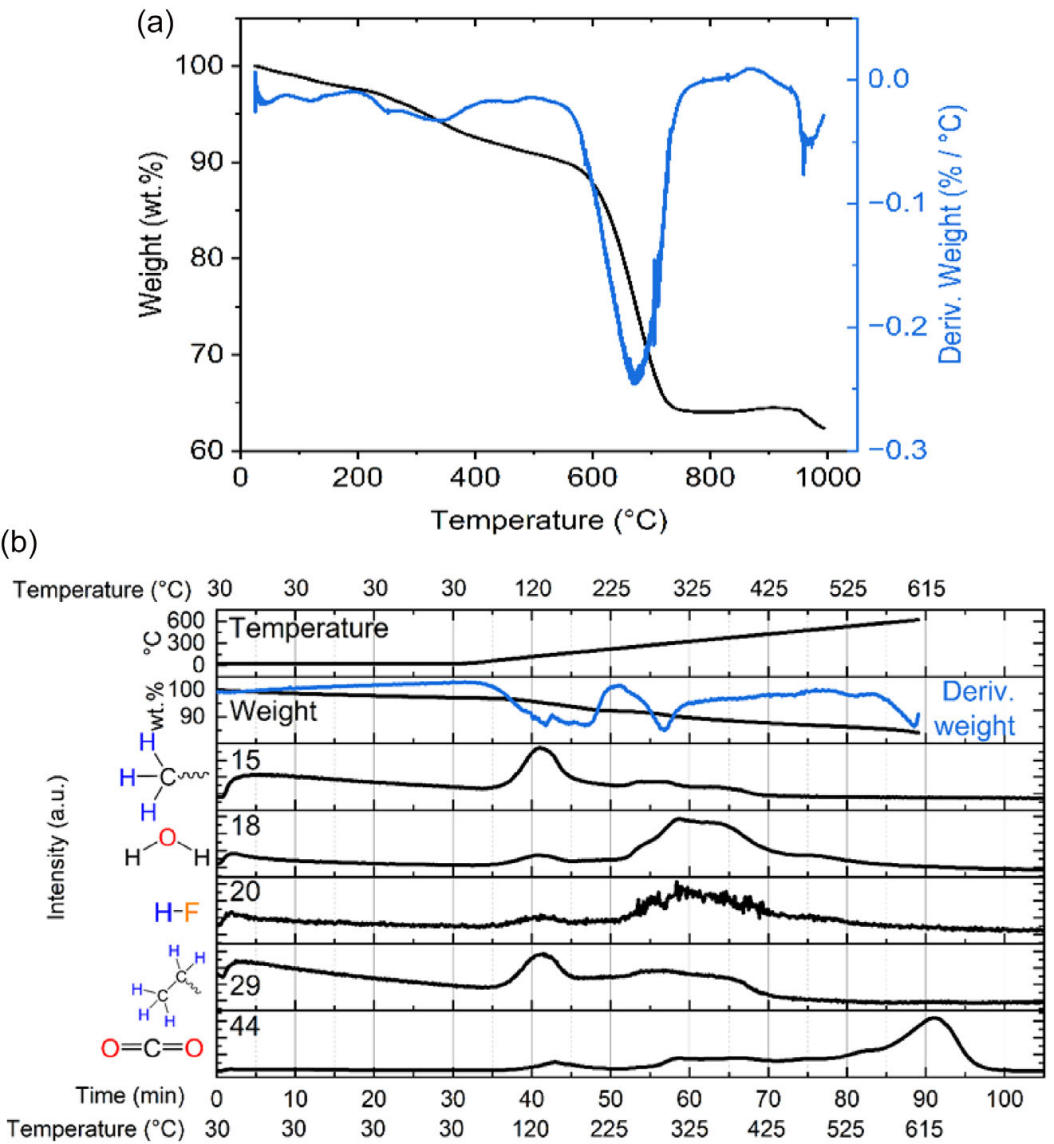
Thermal degradation analysis of the cross‐section sample: a) TGA profile showing sample weight loss (black) and its temperature derivative (blue). b) Coupled TGA–MS analysis presenting the same thermal profile and mass spectrometric signals for selected degradation products.

The thermal degradation of the cross‐section sample in an air atmosphere is a complex process involving multiple decomposition steps. While residual weight at a given temperature is observable, a static thermal weight loss test on a larger scale was also performed due to the dynamic nature of the measurement and the small sample size. The distinct decomposition steps are visible in the temperature‐weight derivative curve shown in **Figure** **2**a. The first significant weight loss, characterized by a peak around 120 °C, is followed by a double peak with maxima around 250 °C and 350 °C. A further small weight loss peak was observed around 450 °C. The most intensive weight loss occurred between 550 and 750 °C. High‐temperature weight loss (above 900 °C) was also observed, which might be connected to fluorine release, as described in work by Ross et al.^[^ ^41^ ^]^

While TGA provides valuable insight into the thermal degradation behavior of materials, it is insufficient to identify decomposition products. Therefore, TGA‐MS was used to detect and identify evolved gases. The results in Figure 2b include the TGA temperature profile, followed by the weight loss curve and its first derivative in the second row. The subsequent rows display MS signal intensities for selected mass‐to‐charge (m/z) ratios: 15, 18, 20, 29, and 44, chosen for their relevance to degradation pathways and evolved moieties. Ion 15 corresponds to the methyl moiety, 18 to water, 20 to HF (though it may also arise from water containing the ^18^O isotope), 29 to ethyl, formyl, or carbon monoxide, and 44 to CO_2_. Water, HF, and CO_2_ were selected as indicative degradation products of the PVDF binder.^[^ ^41^, ^42^ ^]^ HF also originates from the thermal breakdown of the LiPF_6_ electrolyte. Methyl and ethyl groups are typically associated with carbonate‐based electrolyte solvents or with the thermal degradation of the PE separator. Additionally, CO_2_ is a known oxidative degradation product of graphite. However, due to the mass spectrometric resolution of 1 m z^−1^ unit, coeluting or overlapping signals from other fragments cannot be excluded. For the same reason, CO was omitted from interpretation as its nominal mass is the same as N_2_ in the carrier gas.

Decomposition of the cross‐section sample is first indicated by the emergence of peaks for methyl and ethyl groups around 120 °C, associated with the evaporation of carbonate‐based solvents, most likely dimethyl or diethyl carbonate and methyl ethyl carbonate. A broad signal range for methyl, water, hydrogen fluoride, and ethyl between 225 and 450 °C reflects the decomposition of lithium alkyl carbonates.^[^ ^43^ ^]^ The water signal appears as a doublet, with a second local maximum at ≈375 °C, corresponding to the thermal degradation of the PE separator. This is accompanied by ion 15, indicating CH_3_ moieties released during the cleavage of PE chains. Water peak tails extending up to 530 °C are likely attributed to the decomposition of the PVDF binder. At temperatures exceeding 500 °C, a broad CO_2_ signal is observed, corresponding to the thermo‐oxidative degradation of the graphite anode.

Similar TGA–MS measurements were performed on individual anode, cathode, and cathode‐with‐separator samples. The obtained profiles exhibited comparable trends with sample‐specific variations; the cross‐section sample profile (see Figure 2b), however, provides a representative overview and serves as the primary reference for interpretation.

The anode sample exhibited broad peaks of water, hydrogen fluoride, methyl, and ethyl groups centered around 350 °C, primarily from decomposing lithium alkyl carbonates.^[^ ^43^ ^]^ The tailing of water and hydrogen fluoride signals, absent for methyl and ethyl ions, is likely associated with the degradation of the PVDF polymeric binder, similarly to what was observed for the cross‐section sample. The release of carbon dioxide, attributed to the thermo‐oxidative degradation of graphite, began around 500 °C. As pristine graphite is known for its high thermal stability, the observed shift suggests that degradation during battery operation may reduce this stability.

The cathode and cathode‐with‐separator samples displayed behavior with notable differences from the cross‐section and anode specimens. In the cathode‐with‐separator sample, the signals for methyl and ethyl groups appeared as distinct doublets, with lower‐temperature peaks (around 200 °C) attributed to PE degradation. These peaks were less prominent in the cross‐section data, likely due to their relatively low intensity and overlap with the end of carbonates evaporation. For the same sample, the water and carbon dioxide intensities peaked around 375 °C and were noticeably elevated, suggesting ongoing degradation of the PE separator. Both cathode‐based measurements exhibited broad carbon dioxide peaks around 325 °C and 550 °C, even without graphite. These observations indicate that complex reactions may occur within the cathode layers.^[^ ^44^ ^]^ Avoiding cathode exposure to temperatures above 500 °C is recommended to minimize these undesirable decomposition processes.

### 3.2 Static Thermal Mass Loss Analysis

**Figure 3:**
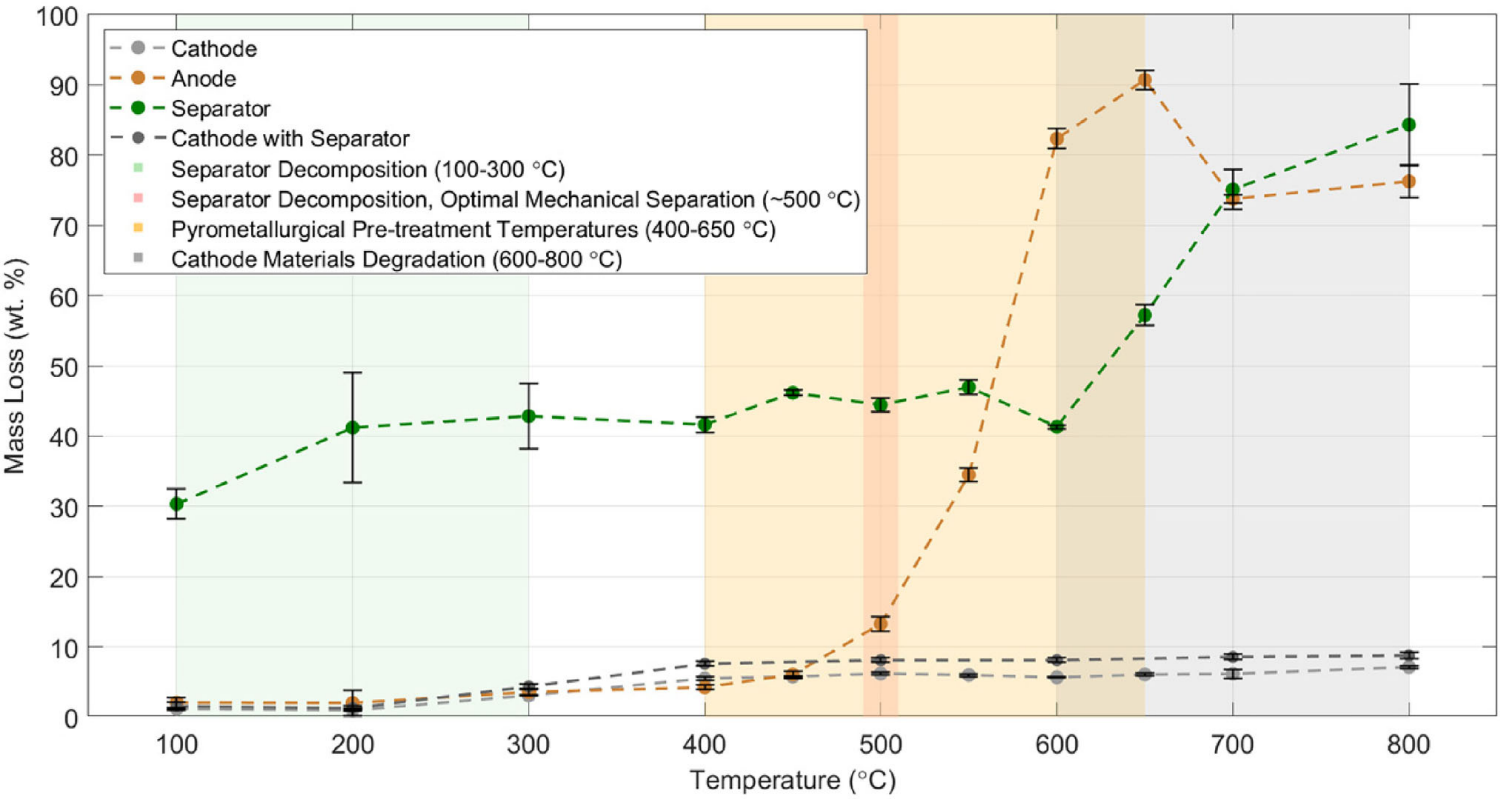
Static thermal mass loss analysis of lithium‐ion battery cell components (cathode, anode, separator, and cathode with separator) from 0–800 °C, illustrating their distinct thermal stabilities and highlighting key decomposition and processing temperature ranges with standard deviation error bars. Note that data points for the cathode with separator sample at 450, 550, and 650 °C are linearly interpolated for visual trend continuity.

Understanding individual LIB components’ thermal decomposition behavior and residual mass is crucial for designing efficient and safe recycling processes. Therefore, their thermal degradation patterns were assessed by monitoring mass loss after controlled heating steps. **Figure** **3** illustrates the components’ comprehensive mass loss profiles across a broad temperature range (0–800 °C). An initial minor mass loss, observed in all samples below 100 °C, is primarily attributed to the evaporation of residual electrolyte. Following this, significant thermal degradation of organic components commences in the separator, predominantly evidenced by mass loss between 100 and 300 °C. This temperature window represents the primary thermal degradation of the separator's polymer matrix. While the polymer (e.g., polyethylene) decomposes at these lower temperatures, the separator's alumina coating remains thermally stable throughout the entire process. By ≈500 °C, these organic constituents were almost entirely decomposed, although minor residues of decomposition products persisted, as confirmed in studies by Mouse et al.^[^ ^45^ ^]^ and Sadeghi and Restuccia.^[^ ^46^ ^]^

The anode exhibits minimal mass loss up to ≈500 °C, after which a sharp and substantial mass loss is observed, indicative of the decomposition of its organic binders and residual carbonaceous materials. Conversely, the cathode and the cathode with separator demonstrate high intrinsic thermal stability, with low mass loss recorded across the 100–800 °C range.

Quantitative analysis of these mass loss profiles provides critical insights into optimal thermal pretreatment strategies for LIB recycling. The pronounced mass loss events observed at specific temperatures correlate strongly with underlying decomposition processes within the battery components. A temperature of ≈500 °C was identified as an optimal upper limit for preserving material integrity and enabling efficient mechanical separation; this temperature balance is crucial for energy‐efficient processing and reducing potential hazardous emissions. At this point, the separator is transformed mainly into refractory Al_2_O_3_, which may facilitate targeted surface functionalization, and enhances the efficiency of downstream metal recovery by minimizing impurities.^[^ ^33^, ^40^ ^]^

By defining a precise temperature of 500 °C for pyrometallurgical pretreatment, this study offers a significant advantage over processes operating at higher temperatures. Compared to conventional pyrolysis methods that often require temperatures of 700 °C or higher for complete organic removal,^[^ ^40^ ^]^ our findings suggest a substantial potential for energy savings and reduced processing time. This approach not only reduces energy consumption but also mitigates the risk of current collector melting and other side reactions that compromise material quality, thereby providing a more efficient and sustainable pathway for battery recycling.

To further characterize the precise thermal behavior of key components within the most critical range for pyrometallurgical pretreatment,^[^ ^45^, ^47^ ^]^ mass loss observations were focused within the 400 to 650 °C temperature range. This window specifically captures the onset and progression of significant mass loss from the anode and the final stages of separator degradation, which are crucial for defining optimal pretreatment conditions. It is important to note that for the 'Cathode with separator’ sample, data points at 450, 550, and 650 °C were not experimentally determined, and its trend line in Figure 3 is linearly interpolated for visual continuity. Conversely, exposure to higher temperatures between 600 and 800 °C led to oxidation and structural degradation of metallic components, particularly the Al current collector in NMC cathodes. This degradation was found to reduce material recovery efficiency and compromise the quality of recovered fractions.

### 3.3 Morphological and Elemental Composition Changes Analysis

**Figure 4:**
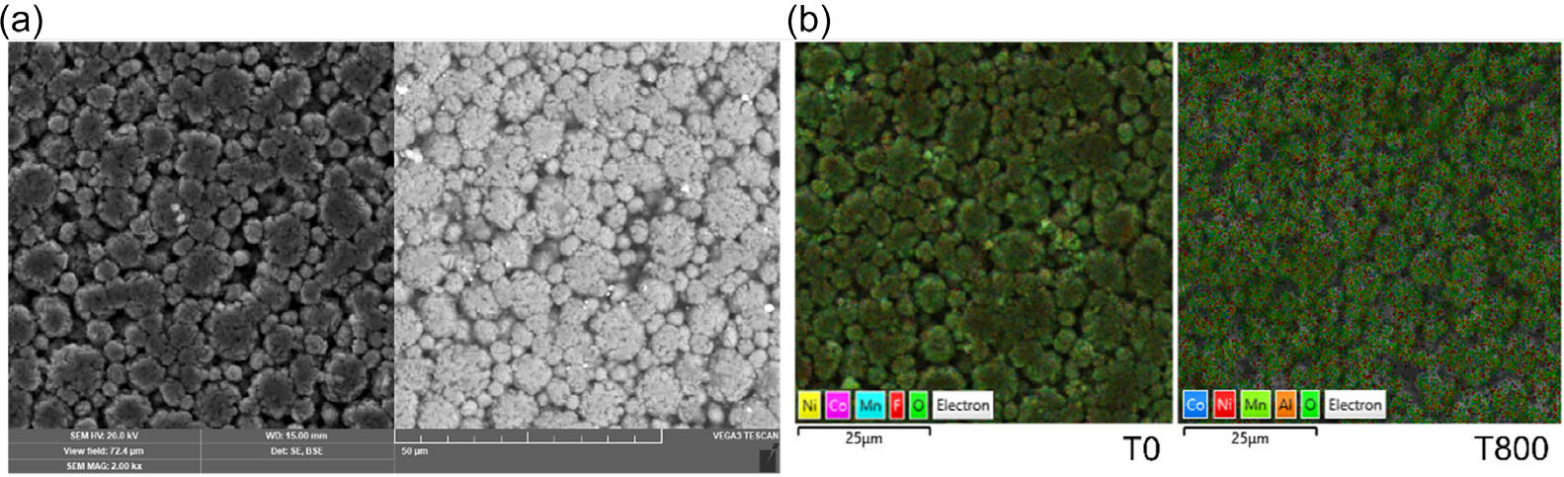
Illustration of a) SEM measurements for the untreated cathode sample using secondary electrons imaging ‐ left, backscattered electrons imaging ‐ right; b) EDS mapping for the untreated cathode sample (T0) compared to the thermally treated cathode sample at 800 °C (T800). 'T’ denotes the specific temperature at which the analysis was performed.

**Figure 5:**
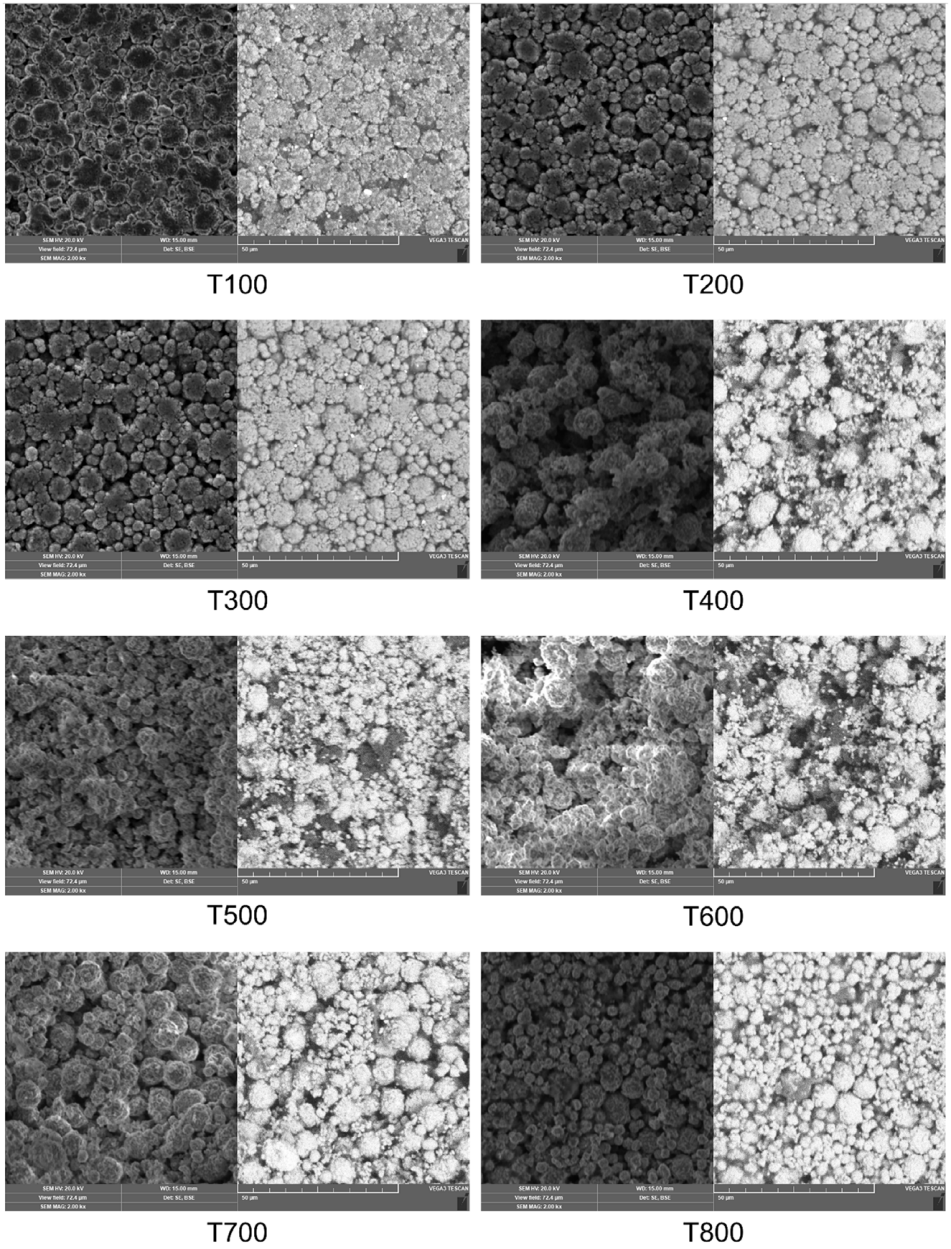
Illustration of SEM measurements for the cathode sample across a temperature range of 100–800 °C, with increments of 100 °C, using secondary electrons imaging ‐ left, backscattered electrons imaging ‐ right. ‘T’ denotes the specific temperature at which the analysis was performed.

Building upon the insights from thermal mass loss analysis, further investigation was conducted into the structural and compositional stability of the cathode materials. In line with the recycling objectives that prioritize the recovery of valuable metals found primarily in battery cathodes,^[^ ^38^, ^48^ ^]^ thermally exposed cathode samples were analyzed to examine their morphological and structural changes using SEM and EDS. **Figure** **4** illustrates the representative initial condition of the dried cathode before thermal treatment, along with elemental maps depicting both the baseline state and the state after exposure to the highest temperature achieved (800 °C). This approach was employed to evaluate the surface composition of the cathode and to track the compositional changes resulting from heat treatment across a temperature range of 100 to 800 °C, with increments of 100 °C. The comprehensive results for all exposed cathode samples are provided in **Figure** **5**.

The findings from SEM imaging confirmed preservation of the spinel structure and particle morphology for the investigated NMC layer even after exposure to temperatures up to 800 °C, indicating its exceptional thermal stability. However, a critical observation was the onset of melting of the Al current collector at 700 °C. This melting event significantly compromises the integrity of the surrounding electroactive material by causing delamination and potential encapsulation, thereby diminishing the quality of the recovered NMC spinel and increasing the complexity for subsequent purification. The ultimate objective of the thermal treatment process is to achieve a temperature regime that maintains the original properties of the valuable electroactive materials while effectively removing all organic components and facilitating their subsequent separation from current collectors, supporting high‐value material recovery.

**Figure 6:**
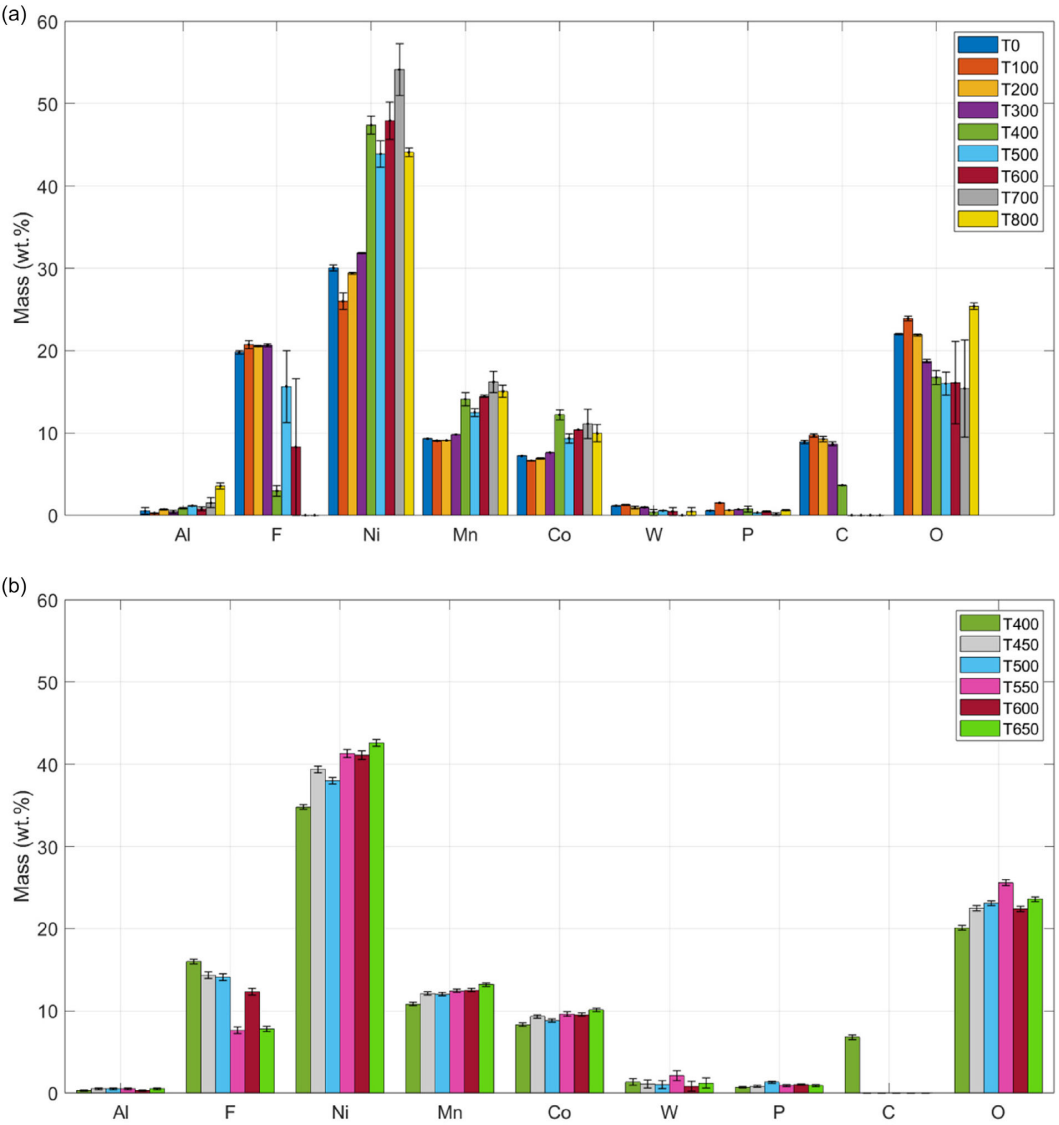
EDS analysis of cathode surface composition after thermal exposure at selected temperatures: a) full range from 0 to 800 °C in 100 °C increments; b) detailed range from 400 to 650 °C in 50 °C increments. ‘T’ indicates the specific temperature at which each sample was analyzed.

Complementing the morphological observations, detailed elemental analysis of the thermally treated cathode surfaces was performed using EDS. The study primarily focused on key electroactive elements (Ni, Mn, Co) and tracking changes in carbon and fluorine residues, as presented in **Figure** **6**. Beyond these primary targets, various other elements originating from auxiliary battery materials were also identified. The detected Al signal is attributed primarily to Al_2_O_3_ residues from the separator,^[^ ^33^ ^]^ which contaminate the cathode surface during disassembly and handling. A minor contribution from the underlying Al current collector foil is also possible in areas where the active material coating is porous or thin. In contrast, Cu was not detected, which is expected as it is the current collector for the anode, and this analysis focused exclusively on the cathode. Phosphorus (P) was identified as a residue from the decomposition of the LiPF_6_ electrolyte salt.^[^ ^20^, ^21^ ^]^ Furthermore, tungsten (W). The presence of W, detected at ≈1 wt%, is attributed to tungsten oxide (WO_3_). WO_3_ is commonly used by battery manufacturers as a surface coating or stabilizing agent for NMC active materials, particularly for Ni‐rich chemistries such as the NMC622 in our study.^[^ ^49^, ^50^ ^]^ This protective layer is known to enhance structural stability and limit degradation from side reactions with the electrolyte, thereby improving the battery's operational lifespan and safety^[^ ^51^, ^52^, ^53^ ^]^ The amount detected by our surface‐sensitive EDS analysis is consistent with levels reported for such functional coatings. Their identification provided insights into potential contamination sources and the completeness of organic removal following thermal treatment. It is important to clarify that Cu was not detected, as it is the current collector for the anode, while this analysis focused exclusively on the cathode. The detected Al signal is attributed primarily to Al_2_O_3_ residues from the separator, which contaminate the cathode surface during disassembly and handling. A minor contribution from the underlying Al current collector foil is also possible in areas where the active material coating is porous or thin.

A critical compositional shift was observed at ≈400 °C, manifesting as a substantial reduction in organic carbon and fluorine content. These changes led to a relative increase in NMC metal content (Ni, Mn, Co) due to the elimination of organic components, as evident from the graphs showing a significant increase in the relative yield of target elements from 400 °C. Notably, at 500 °C, the carbon signal was absent, confirming complete removal of the organic matrix without observable degradation of the NMC phase. Further investigation into narrower temperature intervals revealed that residual carbon was fully combusted even at 450 °C with 1 h of exposure. However, complete fluorine removal was only achieved at higher temperatures, ≈700 °C, indicating the presence of more thermally stable fluorine‐containing compounds at 500 °C. The persistence of these fluorides at 500 °C is a critical consideration, as they can complicate subsequent hydrometallurgical leaching and may contribute to equipment corrosion or lower final product purity. Regarding other elements, residual P from the electrolyte remained unchanged, while oxygen showed an opposite trend, growing at the highest temperatures due to high‐temperature oxidation. Among the identified contaminants, only refractory Al_2_O_3_ and WO_3_ persisted throughout the entire heat treatment. Despite the incomplete fluorine removal at 500 °C, this temperature was reaffirmed as optimal for pyrometallurgical pretreatment, as it effectively combines extensive organic purification (especially carbon removal) with excellent preservation of the electroactive material structure before significant current collector degradation.

The systematic analysis of electrode materials subjected to high‐temperature exposure for 1 h across a range of temperatures from 100 to 800 °C, explicitly focusing on NMC cathodes, graphite anodes, and polymeric separators, has yielded valuable insights into their thermal behavior. The most significant changes in mass and composition were consistently observed between 400 and 650 °C. This comprehensive investigation underscores the suitability of high‐temperature pyrometallurgical approaches for recovering and recycling electroactive materials from spent batteries.

At 100 °C, electrode materials undergo thorough drying to a constant mass. Subsequent heating to 200 °C initiates the oxidation and browning of PE within the separator, alongside the decomposition of PVDF binder. At 300 °C, the oxidation of the separator polymer progresses, accompanied by the liberation of fluorine compounds. The first significant changes in relative composition are observed at 400 °C, primarily marked by the onset of carbon combustion. By 500 °C, all organic carbon is eliminated; however, this temperature also initiates the oxidation of the anodic graphite. The most pronounced oxidation of graphite on the anode occurs at 600 °C, while cathodic layers remain stable. Concurrently, the separator polymer decreases in mass linearly with increasing temperature, and the electroactive material detaches slightly from the current collector. At 700 °C, almost all graphite on the anode is oxidized, but importantly, the Al current collector on the cathode begins to melt, potentially reducing the quality of the NMC spinel. Even at 800 °C, where the Cu current collector of the anode oxidizes and samples disintegrate into powder, it was demonstrated that the NMC spinel crystals largely retain their characteristic structure.

Based on these findings, a temperature of 500 °C has been identified as optimal for the pyrometallurgical separation of electroactive electrode materials. This temperature effectively removes the binder and facilitates straightforward separation of electroactive materials from their current collectors, which remain largely undegraded after just 1 h of exposure. At lower temperatures, the persistence of organic compounds from the electrolyte and binder polymers significantly impedes the separation of electroactive materials and the polymeric separator. Conversely, higher temperatures lead to detrimental oxidative degradation of anodic graphite and metallic current collectors (Al and Cu). While the NMC spinel from the cathode surface remains stable up to 800 °C, its purity and thus the efficiency of separation are compromised at elevated temperatures. The selection of an air atmosphere for this process was a deliberate choice aimed at ensuring the complete removal of organic compounds, which was the primary goal of this pretreatment stage. Although using an inert atmosphere could mitigate graphite oxidation at higher temperatures, such a process would lead to pyrolysis and carbonization of the organic binders and separator polymers. This would result in the formation of a persistent carbonaceous residue (char), which could significantly hinder the efficiency of subsequent recovery steps. Therefore, an oxidative treatment was deemed the optimal approach for producing a clean inorganic material stream, accepting the partial oxidation of the graphite anode at 500 °C as a necessary and manageable trade‐off. Future work could, however, explore a multistep approach, such as an initial vacuum distillation to recover electrolyte solvents before the main oxidative treatment.

## 4 Conclusion

The thermal behavior of key LIB components (NMC cathodes, graphite anodes, and polymeric separators) was systematically examined across a comprehensive temperature range of 100 to 800 °C, with a specific focus on the industrially relevant window of 400 to 650 °C. Unlike prior fragmented studies, an integrated experimental approach was utilized, revealing critical intercomponent interactions and their impact on material integrity during low‐temperature thermal pretreatment for battery recycling. The results show that a treatment temperature of 500 °C offers the best balance for pyrometallurgical pretreatment, allowing complete removal of organic binders and electrolyte residues within 1 h. At this temperature, electroactive materials can be effectively separated from current collectors, which remain mostly intact. Lower temperatures did not eliminate organic carbon compounds, complicating separation, while higher temperatures led to oxidation of graphite and mechanical degradation of collectors. A critical consideration identified in this study is the persistence of fluorine‐containing compounds at temperatures up to 700 °C, which could lead to complications in subsequent hydrometallurgical processing. Despite the thermal stability of NMC cathodes up to 800 °C, their purity decreases at higher temperatures due to interactions with molten current collectors. The findings confirm that treatment within this specific temperature range is favorable for preserving active materials while enabling efficient removal of organics, thereby providing practical insight for the development of energy‐efficient, sustainable, and cost‐effective LIB recycling processes.

The findings of this study also hold significant implications for the sustainability of the LIB recycling chain. By identifying 500 °C as an optimal temperature, our approach offers substantial energy savings compared to conventional pyrolysis or calcination methods that often require temperatures of 700 °C or higher for complete organic removal. This lower energy demand reduces both operational costs and the carbon footprint of the recycling process. From an environmental and material preservation perspective, operating below the melting point of the Al current collector is critical. Our process avoids the collector degradation seen at higher temperatures, which simplifies the separation of high‐purity active materials and prevents the encapsulation of valuable metals. These combined benefits—lower energy use, simpler separation, and purer recovered products—enhance the overall economic viability of the recycling process, providing a practical strategy that strongly supports the implementation of circular economy principles for critical raw materials.

Acknowledge the potential drawbacks, such as the persistence of fluorides at 500 °C, which can complicate subsequent hydrometallurgical leaching and may contribute to equipment corrosion or lower final product purity. This study provides a foundational proof of concept corresponding to a technology readiness level (TRL) of 3–4. While it establishes the optimal temperature and offers a detailed understanding of material transformations on a laboratory scale, we acknowledge that full‐scale validation is necessary to assess industrial process efficiency and scalability. Transitioning from gram‐scale samples to kilogram‐level batches will introduce new engineering considerations. These include challenges in heat transfer, requiring longer residence times or optimized reactor designs to ensure uniform heating, as well as in mass transfer, necessitating an effective purging system to remove gaseous decomposition products and prevent potential side reactions. However, the fundamental decomposition parameters identified in this work are expected to provide a robust basis for these future pilot‐scale studies. Future work should therefore focus on these scalability challenges, assess the performance of recovered materials in reassembled cells, examine gas emissions at a larger scale, and investigate the challenges of incomplete lithium recovery at lower temperatures to bridge the gap between these laboratory findings and industrial implementation. Furthermore, extending this research to other prevalent cathode chemistries, such as NMC532 and high‐nickel NMC811, will be crucial for a comprehensive understanding of low‐temperature pyrometallurgy across the broader LIB market.

This study, therefore, contributes to the basis of the design of thermally driven pretreatment strategies for EOL LIBs. By clearly defining 500 °C as a suitable operational parameter, the work supports the development of energy‐efficient, scalable processes that minimize collector degradation and preserve the electrochemical integrity of active materials. The retention of the NMC spinel structure at this temperature not only facilitates the recovery of high‐purity cathode material but also opens possibilities for direct reuse or simplified downstream refining. These findings advance the understanding of low‐temperature pyrometallurgical processing and offer practical guidance for its implementation in industrial recycling frameworks. As such, this approach supports the broader shift toward circular economy practices in the field of critical raw materials management.

## Conflict of Interest

The authors declare no conflict of interest.

## Authors Contribution

**Anna Pražanová**: resources, methodology, conceptualisation, writing—original draft, project administration. **Jan Kočí**: formal analysis, data curation, visualisation, investigation, writing—original draft, writing—review and editing. **Jonáš Uřičář**: data curation, investigation, visualisation, writing—review and editing. **Dominik Pilnaj**: data curation, investigation. **Daniel‐Ioan Stroe**: supervision, writing—review and editing. **Vaclav Knap**: supervision, writing—review and editing, funding acquisition, project administration.

## Data Availability

The data that support the findings of this study are available from the corresponding author upon reasonable request.
